# Factors associated with locoregional recurrence after neoadjuvant chemotherapy for breast cancer in a safety-net medical center

**DOI:** 10.1007/s10549-025-07668-9

**Published:** 2025-03-04

**Authors:** Danielle Brabender, Deena Hossino, Sean Kim, Margaret Jayich, Lauren Polyakov, David Gomez, Azadeh A. Carr, Stephen F. Sener

**Affiliations:** 1 Department of Surgery, Los Angeles General Medical Center, 1100 North State Street, Clinic Tower 6A231A, Los Angeles, CA USA; 2 Department of Surgery and Norris Comprehensive Cancer Center, Keck School of Medicine of USC, University of Southern California, Los Angeles, CA USA

**Keywords:** Breast cancer, Neoadjuvant chemotherapy, Local recurrence breast cancer, Regional recurrence breast cancer, Axillary sentinel lymph node, Targeted axillary dissection

## Abstract

**Background:**

The management of locally advanced breast cancer poses significant challenges, with contemporary strategies involving an approach that combines systemic and local treatment. The current study was performed to validate the clinical impression that locoregional recurrences have become increasingly uncommon after standardized multimodal treatment protocol.Please check and confirm that the authors and their respective affiliations have been correctly identified and amend if necessary.All authors and affiliations are correct.

**Methods:**

A retrospective analysis was performed using a single-institution database that included clinical, radiographic, and pathologic parameters for all non-metastatic and non-inflammatory breast cancer patients treated with neoadjuvant chemotherapy (NAC) from 2015 to 2023. Uni- and multivariable analyses were performed to define associations between clinical factors, recurrence, and RFS.

**Results:**

The median age was 51 years for 274 predominantly Hispanic (78%) patients, with a median follow-up of 38.1 months. The recurrence rates were 4% local, 2% regional, and 18% distant. Median time from surgery to local recurrence was 8.2 months and to regional recurrence was 9.7 months. There were no locoregional clinical recurrences in 92 (34%) patients who had pCR or in 85 (31%) patients who had radiological complete response after NAC. Locoregional recurrences were uncommon > 12 months after surgery. Five of 11 local recurrences occurred in patients who had a poor response to NAC (ypT4b). All 6 patients having regional recurrences had adjuvant radiation therapy, and only 2 occurred in patients who were pathologically node-negative (ypN0) post-NAC.

**Conclusions:**

Favorable responses to NAC were associated with excellent locoregional control rates. Results achieved for predominantly Hispanic patients at a safety net medical center were similar to those reported in prospective, randomized clinical trials.

## Introduction

The management of locally advanced breast cancer poses significant challenges, with contemporary strategies involving an approach that combines integrated systemic and local treatment [1, 2]. Although randomized trials have demonstrated the advantages of neoadjuvant chemotherapy (NAC), there have been conflicting published results regarding the risk of locoregional recurrence [2]. A meta-analysis of 10 trials using NAC done from 1983 to 2002 demonstrated a 10-year local recurrence rate of 15.1% in those receiving NAC versus 11.9% in those receiving only adjuvant chemotherapy [3]. More recently, results from the I-SPY2 trial and several single-institution reports demonstrated lower risks of locoregional recurrence and no differences after breast-conserving surgery versus mastectomy [4–6]. In these studies, the risk of locoregional recurrence was associated with clinical and pathologic features (including tumor biomarkers), response to NAC, and younger patient age.

Prior data published from this Center on 102 patients with pre-treatment biopsy-proven axillary lymph node metastases treated with neoadjuvant chemotherapy (NAC) from 2015 to 2020 demonstrated that pathologic complete response (pCR), node-negative status after NAC, and triple-negative biomarker subtype were associated with relapse-free survival (RFS) [7]. In that study 56% of patients were node-negative on frozen section and had wire-directed sentinel lymphadenectomy (WD SLND) alone, whereas 43% were node-positive and had WD SLND plus axillary lymph node dissection (ALND). Axillary recurrence was very uncommon after WD SLND alone, making it unlikely that those patients would have derived clinical benefit from the addition of completion ALND to WD SLND.

The current study was performed to contribute insights into the optimal surgical management of locally advanced breast cancer in a largely Hispanic population and to ascertain which clinical-pathologic factors had a discernible impact on locoregional control, recurrence, and survival.

## Methods

A retrospective analysis was performed at a large urban safety net medical center using a single-institution database that included clinical, radiographic, and pathologic parameters for all newly diagnosed non-metastatic and non-inflammatory breast cancer patients treated with NAC between 2015 and 2023. Cancers were clinically staged prior to NAC using the 7th edition (before January 2018) or 8th edition (after January 2018) of the American Joint Committee on Cancer TNM Staging System [8, 9]. The T-category was defined as the largest dimension of the tumor on the clinical, mammographic, ultrasound, or MRI examinations. Pre-treatment scans were performed to exclude metastatic disease (M0) for patients with ≥ T3 tumors or suspicious regional lymph nodes by clinical or radiographic examination. Patients were not restaged after neoadjuvant treatment unless there was a clinical suspicion for disease progression.

Axillary lymph nodes were routinely evaluated by pre-treatment ultrasound, and abnormal nodes were submitted to core needle biopsies and placement of a microclip. All patients in this study then received NAC as recommended in a weekly multidisciplinary breast conference. Patients were generally advised to have chemotherapy if they had ≥ T2 cancers, biopsy-proven axillary lymph node metastases, or preferred breast conservation but had cancers for which mastectomy was more appropriate. The patient-specific treatment plans adhered to regimens for NAC described in National Comprehensive Cancer Network (NCCN) guidelines, based on the cancer stage and tumor biomarkers [10].

Wire-directed sentinel lymph node dissection (WD SLND) was done for patients with pre-treatment biopsy-proven axillary lymph node metastases who received NAC and were clinically node-negative (ycN0) post-NAC. Wire-localization of the metastatic lymph node containing the microclip was done on the day prior to operation using a flexible radial wire. A rotated cranio-caudal mammogram view including the axilla was then done to demonstrate the relationship between the wire and the clipped lymph node. Periareolar injection of technetium 99 m sulfur colloid and lymphoscintigram were also done on the afternoon before operation. Methylene blue dye was injected in the dermis overlying the tumor if the patient did not map by either lymphoscintigraphy or use of the gamma probe to evaluate the axilla in the operating room after the induction of anesthesia, or if the patient was enrolled in a clinical trial which required the use of dual mapping agents.

The WD SLND was done in such a way as to limit the resected lymphadenectomy specimen, as much as possible, to the radioactive (“hot”) and wire-localized (“clipped”) lymph node(s). Lymph nodes removed during WD SLND were imaged in the operating room using a Faxitron™ OR specimen Radiography System (Hologic®, Inc, Mississauga, ON, Canada) to assure that the node containing the microclip was removed. Lymph nodes were evaluated on frozen section by hematoxylin and eosin. Patients who were ypN0 on frozen section had WD SLND alone, while those with pathologically positive nodes (ypN +) on frozen section had WD SLND followed by ALND. ALND was also done for patients who did not map, those whose microclip was not retrieved in the WD SLND, or those who had residual palpable adenopathy identified at the time of axillary surgery.

Post-mastectomy reconstruction was deferred until the completion of all cancer-directed treatment except adjuvant endocrine therapy. For patients with pre-NAC biopsy-proven axillary metastases, adjuvant radiation was considered for the regional lymph nodes, including undissected axillary, supraclavicular, and internal mammary lymph nodes. Whole breast irradiation was delivered to patients after breast-conserving procedures, and chest wall radiation was considered after mastectomy for those with ≥ T3 tumors.

Adjuvant systemic treatment consisted of endocrine therapy for patients with estrogen receptor (ER) + cancers, and adjuvant human epidermal growth factor receptor 2 (HER2)-directed therapy was continued for patients with HER2 + cancers to complete at least one year of therapy. Additional systemic adjuvant treatment was considered when there was residual cancer remaining after NAC, such as Capecitabine (FDA approval 2017) for triple-negative (ER-PR-HER2-) breast cancers, Trastuzumab emtansine (T-DM1) for HER2 + cancers (FDA approval 2019), and Pembrolizumab for tripe-negative breast cancers according to the Keynote 522 protocol (FDA approval 2022) [11–13]. Tumor biomarkers were not routinely repeated on surgical specimens after neoadjuvant chemotherapy. Post-operative follow-up surveillance was done by routine clinical exams, mammograms and ultrasounds when indicated, and CT-PET/CT scans.

Statistical analyses were conducted using SPSS® v27.0 (IBM® Corp, Armonk, NY, USA) and Excel v.16 (Microsoft® Corporation, Redmond, WA, USA). Clinical, radiographic, and pathologic factors known to be associated with survival were included in uni- and multivariable models. Factors with a *p*-value < 0.05 in univariable analysis were submitted to a multivariable analysis, and *p*-values < 0.05 in the multivariable analysis were considered statistically significant. Survival time was calculated from the date of surgical procedure to that of an event, last contact, or death. Events included in relapse-free survival (RFS) were ipsilateral locoregional or distant recurrences of breast cancer. Survival probabilities were estimated using the Kaplan–Meier method and log-rank test. Cox regression was done using the proportional hazards model. The Health Sciences Institutional Review Board at the University of Southern California approved the study (HS-24–00200).

## Results

Descriptive statistics for 274 predominantly Hispanic (78%) patients with non-inflammatory and non-metastatic breast cancer receiving NAC are shown in Table 1. The median patient age was 51 years. There were 119 (43%) ER- and 108 (39%) HER2 + patients, 120 (43%) cancers were multifocal or multicentric, and 188 (68%) patients had pre-treatment clinical stage I-II disease. There were 54 (20%) patients with BMI < 25 (normal), 98 (36%) with BMI 25–30 (overweight), and 122 (44%) with BMI > 30 (obese). The surgical procedure was breast-conserving surgery in 85 (31%) patients and mastectomy in 189 (69%). Adjuvant treatment consisting of chemotherapy was given to 63 (23%) patients, regional node irradiation (RNI) to 172 (63%), and post-mastectomy chest wall (CWRT) or whole breast radiation to 206 (75%).

**Table 1:** Descriptive statistics for 274 patients having neoadjuvant chemotherapy for breast cancer, 2015–2023

Patient factors	N (%)
Age at diagnosis	
< 50 years	133 (49)
> 50 years	141 (51)
Ethnicity	
Hispanic	213 (78)
Asian	25 (9)
African American	10 (4)
White	3 (1)
Other	23 (8)
Focality	
Unifocal	163 (59)
Multifocal	78 (28)
Multicentric	42 (15)
Biomarkers	
ER +	155 (57)
ER-	119 (43)
HER2 +	108 (39)
HER2-	166 (61)
Pre-treatment T-category	
T1	30 (11)
T2	156 (57)
T3	62 (23)
T4	25 (9)
Pre-treatment clinical stage	
I	9 (3)
II	179 (65)
III	75 (27)
IV	2 (1)
Surgical procedure	
Breast-conserving surgery	85 (31)
Mastectomy	189 (69)
Post-surgical therapy	
Adjuvant chemotherapy	63 (23)
Regional node irradiation	172 (63)
Post-mastectomy chest wall irradiation	206 (75)

*ER* estrogen receptor, *HER2* human epidermal growth factor receptor type 2

Factors associated with breast-conserving surgery were unifocal tumor (vs. multifocal/multicentric), cT1/T2 (vs. T3/T4b), cN0 (vs. CN +), and pre-treatment clinical Stage II (vs. Stage III). (Table 2) Patient age at diagnosis, biomarker status, and radiographic response to NAC were not associated with the selection of breast procedure. However, biomarker status (ER + vs. ER- and HER2 + vs. HER2-), pre-treatment clinical Stage (II vs. III), and radiographic response to NAC (radiographic complete response (rCR vs. no rCR) were associated with pathologic complete response (pCR) to NAC. (Table 3) Patient age at diagnosis, focality/centricity of tumor, and type of breast surgery were not associated with pCR. Although 50% of HER2 + and 43% of ER- cancers were associated with pCR, only 10% of ER + HER2- patients had a pCR post-NAC.

**Table 2:** Factors associated with choice of surgical procedure for 274 patients receiving neoadjuvant chemotherapy, 2015–2023

Patient factors	BCS (%)	Mastectomy (%)	p-value
Total	85 (31)	189 (69)	
Age at diagnosis			
< 50 years	38 (29)	95 (71)	*0.42*
> 50 years	47 (37)	94 (67)	*Ref*
Focality			
Unifocal	69 (42)	94 (58)	** < *0.001***
Multifocal	12 (15)	66 (85)	*0.22*
Multicentric	3 (7)	39 (93)	*Ref*
Biomarkers			
ER +	47 (30)	108 (70)	*0.79*
ER-	38 (32)	81 (68)	*Ref*
HER2 +	35 (32)	73 (68)	*0.74*
HER2-	50 (30)	116(70)	*Ref*
Pre-treatment T-category			
T1c	13 (46)	15(54)	***0.001***
T2	56 (36)	100 (64)	***0.003***
T3	14 (23)	48 (77)	*0.06*
T4b	1 (5)	20 (95)	*Ref*
Pre-treatment clinical N-category			
cN0	35 (40)	52 (60)	***0.02***
cN +	50 (27)	137 (73)	*Ref*
Pre-treatment clinical stage			
II	70 (39)	109 (61)	** < *0.0001***
III	10 (13)	65 (87)	*Ref*
Radiographic complete response to NAC (MRI)			
Yes	24 (28)	61 (72)	*0.54*
No	61 (32)	128 (68)	*Ref*

*BCS* breast conserving surgery, *NAC* neoadjuvant chemotherapy, *Ref* reference, *ER* estrogen receptor, *HER2* human epidermal growth factor receptor type 2

**Table 3:** Factors associated with pathologic complete response (pCR) for 274 patients receiving neoadjuvant chemotherapy, 2015–2023

Patient factors	N (%)	pCR (%)	*p*-value
Total	274	92 (34)	
Age at diagnosis			
< 50 years	133 (49)	40 (30)	0.21
> 50 years	141 (51)	52 (37)	Ref
Focality			
Unifocal	163 (59)	60 (37)	0.14
Multifocal	42 (15)	10 (24)	Ref
Multicentric	78 (28)	24 (31)	0.40
Biomarkers			
ER +	155 (57)	30(19)	< 0.0001
ER-	119 (43)	62 (52)	Ref
HER2 +	108 (39)	54(50)	< 0.001
HER2-	166 (61)	38 (23)	Ref
ER + HER2-	94 (34)	9 (10)	0.0001
ER + HER2 +	61 (22)	21 (34)	Ref
ER-HER2-	72 (26)	29(40)	0.48
ER-HER2 +	47 (17)	33(70)	0.0003
Pre-treatment clinical stage			
II	179 (65)	68 (38)	0.019
III	75 (27)	17 (23)	Ref
Radiographic complete response to NAC (MRI)			
Yes	85 (31)	58 (68)	< 0.0001
No	189 (69)	34 (18)	Ref

*NAC* neoadjuvant chemotherapy, *pCR* pathologic complete response *Ref* reference, *ER* estrogen receptor, *HER2* human epidermal growth factor receptor type 2

There were 206 (75%) of the 274 patients who had adjuvant CWRT, and 172 (63%) who had adjuvant RNI. Of those expected to receive a recommendation for adjuvant radiation therapy, a significant majority actually received it: 82% of clinically node-positive patients, 74% of those with cT3/T4 tumors, and 88% of patients with post-NAC pathologically positive axillary lymph nodes.

**Fig. 1:**
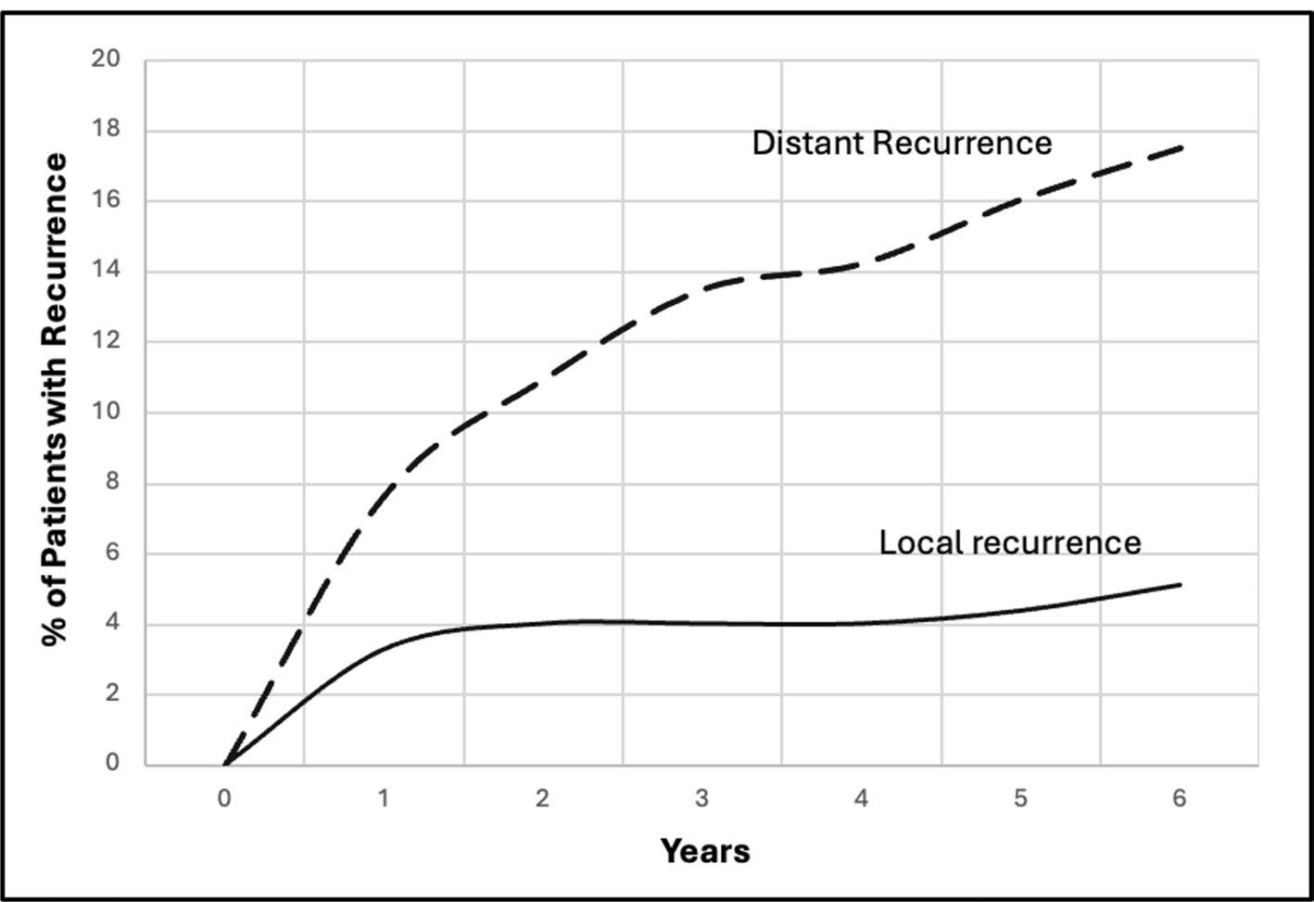
Local and distant recurrences for 274 patients treated with neoadjucant chemotherapy

With median follow-up of 38.1 months, recurrence rates were 4% local, 2% regional, and 18% distant. Median time from surgery to local recurrence was 8.2 months and to regional recurrence was 9.7 months. (Fig. 1) Sixty-two percent of distant recurrences and 78% of local recurrences occurred within two years after surgery. Factors possibly associated with recurrence tested in univariable models were biomarker subtypes, type of surgical procedure, T-category before and after NAC, focality of tumor, response to NAC (ypN status, rCR, pCR), and delivery of adjuvant radiation (RNI and CWRT). In the multivariable analysis, factors related to a favorable response to NAC were significantly associated with low rates of local and distant recurrence. (Table 4) There were no local or regional clinical recurrences in 92 (34%) patients who had pCR or in 85 (31%) patients who had rCR after NAC. Locoregional recurrences were uncommon greater than 12 months after surgery. The local recurrence rates were 4% in 85 patients having breast-conserving surgery and 4% in 189 patients having mastectomy. Five of the 11 local recurrences occurred in patients who had a poor response to NAC (ypT4b). All of the six patients having regional recurrences had adjuvant radiation therapy, and only two occurred in patients who were pathologically node-negative (ypN0) post-NAC.

**Table 4:** Multivariable analysis of factors associated with clinical local and distant recurrences in 274 patients treated with neoadjuvant chemotherapy, 2015–2023

Variable^1^	Total N (%)	Local recurrence *N* = 11 (14%)	Distant recurrence *N* = 48 (18%)
		*p*-value	*p*-value
cT4b	21 (8%)		0.010
cT2	156 (57%)		0.979
ypT2	49 (18%)	0.013	0.002
ypT3	19 (7%)		0.003
ypT4b	6 (2%)	< 0.001	
ER + ER-	155 (57%) 119 (43%)		0.012
HER2 + HER2-	108 (39%) 168 (61%)		0.024
ER + HER2-	94 (34%)		0.058
ypN0	167 (61%)	0.003	< 0.001
pCR	92 (34%)	0.016	< 0.001
rCR	85 (31%)	0.023	< 0.001
RNI	172 (63%)		0.024

^1^The following factors possibly associated with local and distant recurrence were submitted to univariable models: biomarkers, type of surgical procedure, T-category before and after NAC, focality of tumor, response to NAC (ypN0, pCR, and rCR), and delivery of adjuvant radiation (RNI and CWRT). Factors with a *p-*value < 0.05 were tested in multivariable models. Factors remaining significant are in bold

*CWRT* chest wall radiation therapy, *ER* estrogen receptor, *HER2* human epidermal growth factor receptor type 2, *NAC* neoadjuvant chemotherapy, *pCR* pathologic complete response, *rCR* radiographic complete response, *RNI* regional node irradiation

**Fig. 2:**
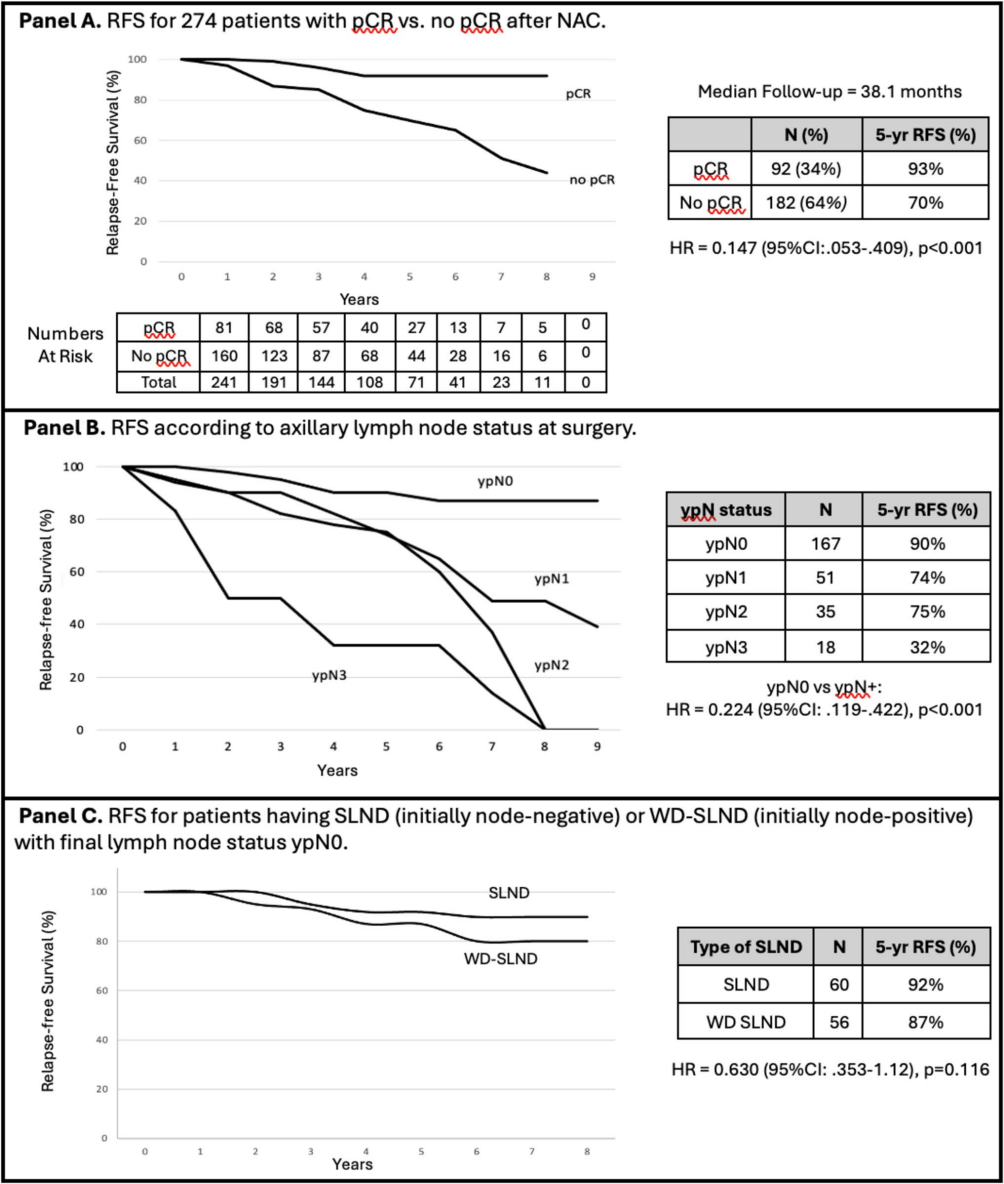
Relapse-free survival (RFS) after neoadjuvant chemotherapy for breast cancer, 2015–2023

Factors associated with relapse-free survival (RFS) were tested in uni- and multivariable models. (Table 5) On multivariable analysis the factors that were significantly associated with RFS were HER2 status, stage of cancer, and the absence of any residual cancer at the time of surgery (pCR, ypN0, or rCR). Patient age, focality of tumor, ER status, and use of postoperative radiation therapy were not associated with RFS. Five-year RFS was significantly higher for the 92 (34%) patients who achieved a pCR versus the 182 (64%) who had not achieved pCR (93% vs. 70%, respectively, HR = 0.147 (95%CI: 0.053–0.409), p < 0.001). (Fig. 2, panel A) Five-year RFS was also significantly higher for patients who were node-negative (ypN0) versus node-positive at surgery (HR = 0.224 (95%CI: 0.119–0.422), p < 0.001). (Fig. 2, panel B) In addition, the 5-year RFS was not significantly different for patients who were cN0 prior to NAC and ypN0 at surgery (92%) versus those who had biopsy-proven axillary lymph node metastases prior to NAC (pN +) and were ypN0 at surgery (87%) (HR = 0.630 (95% CI: 0.353–1.12), p = 0.116). (Fig. 2, panel C) For 29 (40%) of the 72 ER-HER2- patients who developed pCR, the 5-year RFS was 90%, and for the 54 (50%) of 108 HER2 + patients who developed pCR, the 5-year RFS was 100%. On the other hand, for 10% of the ER + HER2- patients who developed a pCR post-NAC the 5-year RFS was 65%, and for the 90% of ER + HER2- patients who did not achieve pCR the 5-year RFS was 67%. For 85 (31%) of the 274 patients who developed rCR post-NAC, the 5-year RFS was significantly higher than for those who did not develop rCR (96% vs. 70%, HR = 0.096 (95% CI: 0.030–0.309), p < 0.001).

**Table 5:** Univariable and Multivariable analysis of factors associated with relapse-free survival for 274 patients receiving neoadjuvant chemotherapy, 2015–2023

Variable^1^	Univariable	Multivariable	
	*p-*value	Hazard Ratio (95% CI)	*p*-value
Age	*0.410*		
Unifocal	*0.770*		
ER +	*0.083*		
HER2 +	*0.007*	0.311 (0.161–0.600)	< *0.001*
ER-HER2-	*0.562*		
ER + HER2-	* < 0.001*	2.841 (1.640–4.924)	< *0.001*
ER-HER2 +	*0.110*		
ER + HER2 +	*0.091*		
Pre-treatment stage IIA	*0.001*	0.298 (0.127–0.697)	*0.005*
Post-treatment stage IIA	*0.658*		
Pre-treatment stage IIIA	*0.658*		
Post-treatment stage IIIA	*0.007*	2.03 (1.098–3.753)	*0.024*
ypN0	* < 0.001*	0.224 (0.119–0.422)	< *0.001*
pCR	* < 0.001*	0.147 (0.053–0.409)	< *0.001*
rCR	* < 0.001*	0.096 (0.030–0.309)	< *0.001*
Post-Op RNI	*0.043*	1.603 (0.853–3.012)	*0.142*
Post-op chest wall radiation	*0.302*		

^1^The factors possibly associated with relapse-free survival were submitted to univariable models. Factors with a *p-*value < 0.05 were tested in multivariable models, and factors remaining significant are in bold

*CI* confidence interval, *ER* estrogen receptor, *HER2* human epidermal growth factor receptor type 2, *pCR* pathologic complete response, *rCR* radiographic complete response, *RNI* regional node irradiation, *ypN0* pathologically node-negative after neoadjuvant chemotherapy

## Discussion

This retrospective study of 274 patients receiving NAC between 2015 and 2023 at a large urban safety net medical center demonstrated that the local and regional recurrence rates were 4% and 2%, respectively, with a median follow-up of 38.1 months. The vast majority of locoregional recurrences occurred within two years after surgery. Multivariable analysis demonstrated that favorable responses to NAC were significant factors associated with low locoregional recurrence and high relapse-free survival rates. In fact, there were no locoregional recurrences in patients who had pCR or rCR post-NAC, and poor responses to NAC were related to a high locoregional recurrence rate. The type of breast surgery was not a factor associated with local recurrence in the current study.

It has been recognized for some time that combined adjuvant systemic and radiation therapy play a role in the locoregional control of disease. Although the American College of Surgeons Oncology Group (ACOSOG) Z0011 and AMAROS trials both enrolled patients with low burdens of disease, they reinforced the principle that adjuvant treatment was associated with reduced rates of locoregional recurrence [14, 15]. It is now apparent that NAC is also associated with decreasing locoregional recurrence rates. In a contemporary report from the I-SPY2 adaptive prospective clinical trial, 1462 patients with breast cancer having high-risk molecular features were randomized to one of several arms of NAC [4]. Even though there was a substantial number of high-risk clinical features in those patients, the locoregional recurrence rate was only 6.3% with median follow-up of 3.5 years. There was no increased risk of locoregional recurrence after breast-conserving surgery versus mastectomy. Triple-negative receptor subtype and increasing extent of residual disease post-NAC were significantly associated with increased risk of locoregional recurrence. These results confirmed prior data from the combined analysis of National Surgical Adjuvant Breast and Bowel Project (NSABP) B-18 and B-27 trials which demonstrated that extent of residual cancer burden was a factor associated with locoregional recurrence [16]. An emerging body of work has since further demonstrated that residual cancer burden was prognostic for long-term survival post-NAC in three phenotypic subsets of breast cancer [17, 18]. The results from the current study were consistent with more recent evidence confirming the relatively low locoregional recurrence rates post-NAC and that response to NAC (pCR, ypN0, and rCR) was a strong factor associated with local recurrence [19, 20]. To put these recurrence rates into historical perspective, in the NSABP B-04 study there were 365 cN0 patients treated with total mastectomy without radiation or systemic therapy, and 18% developed regional node recurrence in the untreated axilla at a median time of 15 months after surgery [21].

The relationship of pCR to RFS in the current study is also consistent with previous reports. In a pooled analysis of 11,955 patients having NAC from 1990 to 2011, the strongest association between pCR and RFS was in patients with aggressive breast cancer subtypes (triple-negative, ER + (grade 3) HER2-, ER-HER2 +) [22]. pCR was achieved in 34% of patients with triple-negative breast cancer, and the 5-year RFS was significantly higher in those who achieved pCR than in those who did not (84% vs. 52%, respectively (HR = 0.24, (95%CI: 0.18–0.33)). On the other hand, only 10% of ER + HER2- patients achieved pCR (7% in grade 1 or 2 and 16% in grade 3 tumors). The 5-year RFS rates for ER + HER2- patients were 88% in those who achieved pCR vs. 75% in those who did not (HR = 0.49, (95% CI: 0.33–0.71)), but the 5-year RFS rates were not significantly different between pCR and no pCR status in ER + HER2- grade 1–2 tumors (HR = 0.63, (95% CI: 0.38–1.04)). In that meta-analysis, increasing rates of pCR did not translate into increasing rates of RFS. In a more recent single-institution retrospective analysis of 1,150 patients treated from 2005 to 2018, rates of pCR were significantly different according to tumor biomarker subtype, ranging from 7.1% in luminal A to 38.1% in triple-negative tumors [23]. Luminal B and triple-negative tumors, cN1 status, age, and lack of pCR retained significant independent adverse prognostic impact in a multivariable analysis. Compared to the results of these two studies, in the current study, biomarker status (ER + vs. ER- and HER2 + vs. HER2-) along with pre-treatment clinical Stage (II vs. III) and radiographic response to NAC (rCR vs. no rCR) were associated with pCR post-NAC. Although 50% of HER2 + and 43% of ER- cancers were associated with pCR, only 10% of ER + HER2- patients had a pCR.

Mirroring these prior results, the multivariable analysis in the current study revealed that the factors significantly associated with RFS were the absence of any residual cancer at the time of surgery (pCR and rCR), node-negative status at surgery (ypN0), HER2 status, and stage of cancer. Five-year RFS was significantly higher for patients who achieved a pCR versus those who had not achieved pCR. (Fig. 2) For patients who achieved pCR, the 5-year RFS was 90% for ER-HER2- and 100% for HER2 + patients. On the other hand, for 10% of the ER + HER2- patients who developed a pCR post-NAC the 5-year RFS was 65%, and for ER + HER2- patients who did not achieve pCR the 5-year RFS was 67%. Furthermore, although ER + HER2- patients received adjuvant endocrine therapy, the relatively low relapse-free survival rates regardless of pCR status amplify the need for the development of new treatment strategies for this group of patients. Another observation worthy of emphasis regarding 5-year RFS was that the axillary lymph node status at surgery (ypN0) was a more important prognostic factor than node status pre-NAC (cN0 or pN +). Based on that observation, it might be tempting to consider omission of ultrasound evaluation for axillary lymph nodes pre-NAC, but the identification and removal of histologically positive lymph nodes (pN +) during SLND has been shown to reduce the false-negative rate of SLND post-NAC [24].

An important strength of this study was its performance at an urban safety net medical center serving a predominantly Hispanic population. Hispanic women are likely to present with advance-stage breast cancer, potentially leading to increased requirements for NAC and to worse outcomes [25]. The original data on NAC were produced in prospective, randomized clinical trials which were under-represented by Hispanic patients. For all breast cancer patients from 2003 to 2016 enrolled in completed therapeutic trials, 82.3% were non-Hispanic whites, 8.*6%* were African Americans, and 5.7% were Hispanics [26]. While the underrepresentation of diverse populations in clinical trials stresses the need for more inclusive research, broader systemic disparities further exacerbate inequities in breast cancer outcomes. These disparities are strongly influenced by social determinants of health, including insurance coverage, socioeconomic status, and access to timely medical care [27, 28]. In addition, variations in clinical and pathologic features of breast cancer across races may also be contributors to disparities in outcomes [29]. A recent ad hoc analysis of clinical outcomes by race and ethnicity was published for women with HR + HER2-, node-positive breast cancer enrolled in the randomized phase III RxPONDER trial [30]. Of 4,048 women with self-reported race ethnicity, 15% were Hispanic, 6% non-Hispanic Black (NHB), 8% Asian, 0.8% Native American/Pacific Islander, and 70% non-Hispanic White (NHW). There were no differences by race/ethnicity for tumor size, number of positive lymph nodes, or Oncotype DX® Recurrence Score results. Notably for 610 Hispanic patients, 28% had BMI < 25 (normal), 35% had BMI 25–30 (overweight), and 38% had BMI > 30 (obese), similar to data for BMI reported in the current study. Compared with NHWs, Invasive Disease-free Survival (IDFS) and Distant Relapse-free Survival (DRFS) were worse for NHB and better for Asians. IDFS and DRFS were similar for NHW and Hispanic patients (5 yr IDFS 91.6% vs. 91.4% and 5-yr DRFS 95.7% vs. 95.3%, respectively). The efficacy of chemotherapy did not differ in this trial by race/ethnicity. Adjusting the analysis for clinical features, especially BMI, reduced the effect of race on outcomes. Although the results of the RxPONDER trial were obtained in the setting of adjuvant systemic therapy, the study demonstrated the importance of including race/ethnicity as potential factors related to survival outcomes.

Until oncology cooperative groups achieve greater diversity in clinical trials and generate more robust results from randomized, prospective trials regarding race/ethnicity, it will remain important to report retrospective studies that include minority populations, such as the predominantly Hispanic population described in the current study [31]. The important role of high-quality safety net hospitals in mitigating healthcare inequities was reinforced in the current study by ensuring that even uninsured or those who had Medi-Cal or Medicaid expansion healthcare plans received timely state-of-the-art breast cancer care. This level of care was delivered despite working through recognized systemic barriers, such as socioeconomic status, language differences, delayed referrals, financial constraints, inadequately addressed medical co-morbidities, and transportation difficulties [32, 33].

This study had several potential limitations. It was a retrospective, single-institution study with potential for selection and observer bias. Although there was a consistent rationale over the course of this study for systemic treatment and adjuvant radiotherapy treatments were standardized by institutional treatment regimens, individual treatment plans differed by patient and these differences were not controlled for in data analysis. Chemotherapy regimens also changed over time to include adjuvant chemotherapy and immunotherapy agents based on completion of recently reported clinical trials and FDA approval of drugs. In addition, differentiation between anthracycline and non-anthracycline regimens was not considered in the analysis.

## Conclusion

With contemporary systemic chemo-endocrine therapy agents and modern radiation therapy techniques, locoregional recurrences rates were under 5% regardless of the choice surgical management of the breast. Favorable responses to NAC were significant factors associated with low locoregional recurrence and high relapse-free survival rates.Please confirm the section headings are correctly identified.Section headings are correct.

## Data Availability

The datasets generated and analyzed for the current study are not publicly available but are available from the corresponding author upon reasonable request.
